# Risk Factors Associated With Epidural Use

**DOI:** 10.4021/jocmr810w

**Published:** 2012-03-23

**Authors:** Samuel M. Lancaster, Ursula M. Schick, Morwan M. Osman, Daniel A. Enquobahrie

**Affiliations:** a Department of Epidemiology, University of Washington, Seattle, Washington, USA; b Department of Public Health Genetics, University of Washington, Seattle, Washington, USA

## Abstract

**Background:**

Identify variables associated with intrapartum epidural use.

**Methods:**

Odds ratios were calculated to quantify associations between selected variables and epidural use using a population-based case-control study of Washington State birth certificate data from 2009.

**Results:**

Non-Whites had 10 - 45% lower odds of epidural use relative to Whites. Foreign-born women had 25 - 45% lower odds of epidural use compared to their US-born counterparts, except for Asians. Women who smoked or induced labor had higher roughly 2-fold higher odds of epidural use compared with non-smokers or women giving birth spontaneously, respectively. Women without a high school diploma or equivalent had lower odds of epidural use relative to those who graduated. Delivering at perinatal units, rural hospitals, or non-profit hospitals had ~50% lower odds of epidural use compared with secondary/teritiary perinatal units, urban hospitals or for-profit hospitals, respectively.

**Conclusion:**

Several individual and health service-related variables were associated with epidural use. These findings elucidate the clinical relevance of epidural use, and dispariaties in its utilization and in quality of care during delivery.

**Keywords:**

Epidural use; Foreign birth; Labor; Racial disparities

## Introduction

Intrapartum pain relief is commonly administered as a central nerve block involving injection of an analgesic into the lumbar region of the lower back. Methods of central nerve blocking for intrapartum pain relief include epidural analgesia, spinal block or combined spinal-epidural, all of which provide effective pain relief while allowing appropriate motor function. In this paper, we will refer to these procedures collectively as epidurals. Intrapartum epidural use is associated with a more comfortable labor and a better overall labor experience [1]. However, epidural use is also associated with an increased risk of instrumental delivery, fetal malposition, longer labor, and fetal distress compared to other forms of intrapartum analgesia (e.g. intravenous opioids) [1-3]. Despite these potential risks, rates of epidural use in the United States and other industrialized countries have increased dramatically in recent years [4-6]. Currently, in the United States, approximately 60% of parturients (delivering women) receive epidurals for intrapartum pain relief [3].

While epidural use is becoming widespread, it nonetheless varies across parturient subgroups. Previous US-based studies have identified associations between epidural use and the following variables: socioeconomic status, parity, plurality, labor induction, maternal age, race/ethnicity, insurance status, and urban residence [7-9]. In non-US based studies, several of these variables (including race, socioeconomic status, parity and age) have also been reported to be associated with epidural use [4, 10-12]. Better understanding associations with epidural use may help elucidate disparities in epidural utilization and underscore the clinical importance of epidurals in certain circumstances.

To validate previously-discovered associations with epidural use and discover new associations, we conducted a population-based case-control study to determine the relationship between epidural use and both individual maternal variables and health service-related variables. Individual variables evaluated were labor induction, smoking, race, education, and foreign birth. Health service-related variables evaluated were hospital perinatal tier, location of hospital, and hospital ownership. Importantly, the association between epidural use and smoking, foreign birth, hospital perinatal tier, and hospital ownership has never been published. Additionally, few large, population-based studies have investigated variables associated with epidural use. Such studies have the advantages of strong statistical power and broad generalizability over smaller studies.

## Materials and Methods

### Study population

We conducted a population-based case-control study using data from Washington State birth certificates that were linked with the Comprehensive Hospital Abstract Reporting System (CHARS) from 2009. CHARS is a resource created by the Washington State Department of Health (WS DOH), and contains hospital discharge records for all nonfederal facilities in Washington State. Additional covariate information was obtained using WS DOH definitions [13, 14] to classify hospitals.

The study population consisted of 43,410 mothers who had singleton live births by vaginal delivery in a Washington State hospital. Randomly sampled epidural users (n = 21,705) were matched to non-users (n = 21,705) in a 1:1 ratio. Epidural use or non-use was determined by the presence or absence, respectively, of a checked response to the question labeled “epidural or spinal anesthesia during labor” in the birth filing form. University of Washington Institutional Review Board exemption was obtained prior to the initiation of this study.

### Defining individual variables and health service-related variables of interest

Individual variables and health service-related variables were ascertained through a combination of responses to questions on the birth filing form, linked CHARS data, and WS DOH definitions. Individual variables investigated for associations with epidural use were induction of labor (induced labor, spontaneous labor), smoking (smoking reported during pregnancy, no smoking reported during pregnancy), race (non-Hispanic White, non-Hispanic Black, Asian, Hispanic), education (no high school degree, high school degree equivalent or greater), and foreign birth (foreign birth, US birth). Induction of labor was ascertained based on the response to a check box (62.1) on the birth filing form. Maternal smoking status was obtained from birth filing form question 39. Maternal race was identified through question 22 of the birth filing form indicating self-identified race and classified in accordance with the National Institutes of Health guidelines [15]. Maternal education was ascertained through question 20 on the birth filing form. Foreign birth status was determined using maternal country of birth (question 8) of the birth filing form. Individuals with listed countries of birth outside of US were considered to be foreign-born.

Health service-related variables of the delivery hospital we investigated as exposures were tier of perinatal unit (primary, secondary/tertiary), location (urban, rural), and ownership type (state, military, region, proprietary, district). The tier of perinatal unit is a measure of the level of care provided at a perinatal facility. Definitions of perinatal tiers were obtained through the Washington State Perinatal Level of Care Guidelines [13] and were linked to birth certificate data through the birthing facility unique hospital identifier. Primary perinatal units are facilities capable of caring for normal, term deliveries with no or minimal complications for the mother or neonate and are capable of performing cesarean delivery and anesthesia within 30 minutes of request. In addition to the capabilities of primary units, secondary perinatal units provide care for selected maternal complications and neonates of 34 weeks gestation and older. Tertiary perinatal units care for infants of all gestational age and have immediate access to cesarean deliveries and anesthesia. The Washington State Department of Health’s Hospital directory was used to classify hospital ownership and setting [14] and was linked to birth crtificate data through the birthing facility unique hospital identifier.

### Statistical methods

Multivariate logistic regression analyses with robust variance estimates were used to generate odds ratios and 95% confidence intervals. Many covariates were investigated as potential confounding factors in the relationship between epidural use and variables of interest. Confounders found to alter odds ratios by 10% or more were adjusted for in the final estimates. Final estimates for individual variables were adjusted for age, insurance provider and delivery hospital ownership. Health service-related estimates were adjusted for age, race, education, induction of labor and insurance provider. Maternal age was adjusted for in all analyses a priori. Race was analyzed as an effect modifier for foreign birth. Adjusted odds ratios were considered to be statistically significant if the 95% confidence interval did not span the null value of 1.00. All analyses were conducted using Stata, version 11.1 [16].

## Results

Table 1 presents the characteristics of the mother and birthing facility by epidural usage status. Distributions of martial status, age and education were similar between epidural users and non-users. However, epidural users and non-users differed with respect to distributions of insurance provider and parity. Epidural users were more likely to be privately insured (50.39%) than non-users (42.85%), and the mean parity of epidural users was lower (0.92 ( 1.24) than non-users (1.33 ( 1.46).

**Table 1:** Characteristics of Epidural Users and non, Users who Gave Birth Vaginally to Singletons in Washington State, 2009

		Epidural users (n = 21,705)	Epidural non-user (n = 21,705)
	**Maternal Variables**		
	**Variable**	**Mean (SD)**	**Mean (SD)**
	
	Maternal Age (years)	27.10 (5.87)	27.94 (5.98)
	Parity	0.92 (1.24)	1.33 (1.46)
	Education (years)	13.28 (2.63)	12.65 (3.18)
	
	**Status**	**n (%)^a^**	**n (%)^a^**

Marital Status	Married	14,034 (65.10)	13,981 (65.10)
	Unmarried	7,523 (34.90)	7,496 (34.90)
Insurance Provider	Government	10,458 (48.92)	11,997 (56.18)
	Uninsured	147 (0.69)	206 (0.97)
	Private	10,773 (50.39)	9,088 (42.85)
Induction of Labor	Spontaneous	15,164 (70.22)	18,174 (84.22)
	Induced	6,430 (29.78)	3,404 (15.78)
Smoking During Pregnancy	Did Not Smoke	18,955 (88.60)	19,900 (92.70)
	Smoked	2,439 (11.40)	1,567 (7.30)
Race	Non-Hispanic White	16,186 (75.72)	13,320 (62.58)
	Non-Hispanic Black	1,030 (4.82)	1,029 (4.83)
	Hispanic	1,713 (8.01)	4,091 (19.22)
	Asian	1,844 (8.63)	2,117 (9.95)
	Other	603 (2.82)	728 (3.42)

	**Hospital Variables**		
	**Location**	**n (%)^a^**	**n (%)^a^**

Perinatal Unit Tier	Primary	4,798 (23.85)	2,879 (15.32)
	Secondary or Tertiary	15,322 (76.15)	15,912 (84.68)
Hospital Location	Urban	16,324 (83.58)	16,502 (91.91)
	Rural	3,208 (16.42)	1,453 (8.09)
Hospital Ownership	Not for Profit	13,534 (68.09)	13,376 (68.83)
	District	5,164 (25.98)	3,671 (18.89)
	For Profit	319 (1.60)	425 (2.19)
	State	514 (2.59)	511 (2.49)
	Military	347 (1.75)	1,478 (7.61)

^a^Percentages may not add to 100 due to rounding. Numbers may not add to total due to missing data.

All individual maternal variables were associated with epidural use after adjustment for maternal age, insurance, and hospital ownership (Table 2, 3). Parturients with induced labor had an adjusted odds ratio (aOR) of epidural use that was 2.16 times that of parturients with spontaneous labor (95% CI (2.06, 2.27)). Relative to non-smoking parturients, those that reported smoking during pregnancy had increased odds of epidural use (aOR = 1.71 95% CI (1.59, 1.84)). Non-White race and lower maternal education were associated with lower odds of epidural use than the referent No-Hispanic White race and higher education, respectively. Asian and non-Hispanic Black women had lower odds of epidural use compared with non-Hispanic Whites (aOR = 0.86, 95% CI (0.79, 0.95); aOR = 0.79, 95% CI (0.73, 0.85), respectively). Hispanic women had the lowest odds of epidural use compared with White women (aOR = 0.37, 95% CI (0.35, 0.40)). Women without a high school degree had lower odds of epidural use compared to those who had a degree (aOR = 0.55, 95% CI (0.52, 0.58)). Foreign birth was associated with epidural use among all racial groups except Asians (Table 3). Relative to US-born women, foreign-born women had reduced odds of epidural use (aOR = 0.51, 95% CI (0.49, 0.53), Table 3). In particular, foreign-born Blacks had the lowest odds of epidural use compared with US-born Blacks (aOR = 0.35, 95% CI (0.29, 0.43)). Foreign-born Whites and Hispanics had lower odds of epidural use compared to US-born Whites and Hispanics (aOR = 0.52, 95% CI (0.49, 0.56); aOR = 0.68, 95% CI (0.59, 0.78), respectively). Interestingly, foreign-born Asians had comparable odds of epidural use as US-born Asians (aOR = 0.96, 95% CI (0.82, 1.12)).

**Table 2:** Odds Ratios of Associations Between Intrapartum Epidural Analgesia Use and Individual Variables in Washington State, 2009

Maternal Factors	Adjusted OR (95% CI)^a^
Induced Labor	
Induced	Referent
Spontaneous	2.16 (2.06, 2.27)
Smoking during pregnancy	
Did Not Smoke	Referent
Smoked	1.71 (1.59, 1.84)
Race	
Non-Hispanic White	Referent
Non-Hispanic Black	0.86 (0.79, 0.95)
Hispanic	0.37 (0.35, 0.40)
Asian	0.79 (0.73, 0.85)
Education	
High school degree or higher education	Referent
No high school degree	0.55 (0.52, 0.58)

^a^Adjusted for maternal age, insurance, and hospital ownership. Robust multivariate logistic regression was used to determine odds ratios and confidence intervals.

**Table 3:** Odds of Epidural Use According to Foreign Birth Status Stratified by Maternal Race in Washington State in 2009

Foreign Birth Stratum	Adjusted OR (95% CI)^a^
All	
US-Born	Referent
Foreign-born	0.51 (0.49, 0.53)
Non-Hispanic White	
US-Born	Referent
Foreign-born	0.52 (0.49, 0.56)
Non-Hispanic Black	
US-Born	Referent
Foreign-born	0.35 (0.29, 0.43)
Hispanic	
US-Born	Referent
Foreign-born	0.68 (0.59, 0.78)
Asian	
US-Born	Referent
Foreign-born	0.96 (0.82, 1.12)

^a^Adjusted for maternal age, insurance, and hospital ownership. Robust multivariate logistic regression was used to determine odds ratios and confidence intervals.

Several health service-related variables were found to be associated with epidural use after adjustment for age, race, education, induction of labor and insurance provider (Table 4). Parturients delivering in secondary or tertiary perinatal unit had higher odds of epidural use (aOR = 1.61, 95% CI (1.52, 1.70)). Delivering in a rural hospital was associated with lower odds of epidural use (aOR = 0.48, 95% CI (0.44, 0.51)). Higher odds of epidural use were observed for for-profit and military hospitals (aOR = 1.37, 95% CI (1.18, 1.61); aOR = 5.29, 95% CI (4.67, 6.00), respectively) compared with private not-for-profit hospitals. Conversely, district hospitals had slightly lower odds of epidural use (aOR = 0.78, 95% CI (0.74, 0.82)). No difference in epidural use was observed between state hospitals and private not-for-profit hospitals (aOR = 1.08, 95% CI (0.94, 1.23)).

**Table 4:** Associations of Health Care Related Factors With Intrapartum Epidural Analgesia Use in Washington State in 2009

Health Care Related Factors	Adjusted OR (95% CI)^a^
Perinatal Unit Tier	
Primary	Referent
Secondary or Tertiary	1.61 (1.52, 1.70)
Location	
Urban	Referent
Rural	0.48 (0.44, 0.51)
Ownership	
Not for Profit	Referent
District	0.78 (0.74, 0.82)
For Profit	1.37 (1.18, 1.61)
State	1.08 (0.94, 1.23)
Military	5.29 (4.67, 6.00)

^a^Adjusted for maternal age, insurance, induction, race and education. Robust multivariate logistic regression was used to determine odds ratios and confidence intervals.

## Discussion

In this large, population-based case-control study, several individual maternal variables (induction of labor, smoking status, race, education and foreign birth) and health service-related variables (perinatal tier, rural/urban setting, and hospital ownership type) were associated with epidural use. Blacks, Hispanics and Asians had lower odds of epidural use compared with Non-Hispanic Whites. In addition, smokers, US born mothers, ≥12 grade educated mothers, and those who had induced labor had higher odds of epidural use compared with their respective counterparts. Giving birth at a secondary/tertiary perinatal unit, giving birth at for-profit or military hospitals, or giving birth in an urban setting was associated with higher odds of epidural use compared with primary perinatal unit, private non-profit hospital or rural setting, respectively.

To the extent to which disparities exist between clinical practices and reporting on birth filing and hospital forms, we expect a similar degree of misclassification. This problem is particularly acute during labor when the number of procedures is often under-reported [17]. Furthermore, birth certificates are routinely filled out as administrative data and are not specifically designed for epidemiological studies, which may contribute to misclassification in this study.

Our analysis was also limited by the imprecise parsing of race classifications provided by CHARS discharge data and Washington State birth certificates. Epidural usage varied widely between foreign country; however, we were unable to compare individuals from these countries to their US-born counterparts as US ancestry information is not collected. To alleviate this problem, future studies would need to assess ancestral origin as well as use more precise racial categories.

In addition, this study is limited by heterogeneity in outcome and epidural non-user definitions. The outcome, epidural use, is a heterogeneous classification including both epidural analgesia and spinal anesthesia. Distinguishing between these forms of analgesia may change risk estimates. Beyond this limitation, we did not investigate use of alternative methods of pain relief. This omission introduces heterogeneity in the epidural non-user population by including both women who used alternative pain relief and those not using any form of pain relief. Therefore we may have some confounding by indication. We recognize that if we were able to resolve these issues, our risk estimates might change.

Induction of labor was found to be strongly associated with epidural use. This finding is consistent with previously reported results. A US-based study found similar, but not statistically significant, higher odds of epidural use associated with induction of labor (OR = 2.30, 95% CI 0.50 - 9.40) [9]. Further, an Australian study reported higher odds of epidural use among women with induced labor, similar to our report (OR = 1.86, 95% CI 1.83 - 1.88) [18]. Induction of labor is likely associated with epidural use for several reasons. First, induced labor is often more painful than spontaneous labor. This increase in pain is indicated by the increase in epidural dosage among women with induced labor [19]. Secondly, labor is induced more often when there are complications, and complications themselves are associated with epidural use [20]. Finally, we speculate women who elect to have labor induced are likely more comfortable with other medical interventions, such as an epidural, further contributing to the observed association between induction of labor and epidural use.

To our knowledge, this is the first study to look at the association between epidural use and smoking. Smoking is associated with an increase in complications during pregnancy, which are themselves associated with epidural use [21]. Further, smoking is a latent variable for other potential factors, such as impulse control and stress levels. These latent variables might themselves be responsible for the observed association of smoking with epidural use.

In this study, we reported that not obtaining a high school degree or equivalent was associated with lower odds of epidural use. Using education level as an indicator of socioeconomic status, we can infer that low socioeconomic status may be associated with lower odds of epidural use. These findings are consistent with a previous study reporting that women from the poorest quintile had a lower likelihood of epidural use than those from the richest quintile (OR = 0.59, 95% CI 0.58, 0.61) [3, 4]. The mechanism for this association is unknown, but may be related to cultural practices or reduced access to resources.

Previous studies have also reported that being non-Hispanic Black, Asian or Hispanic was associated with lower odds of epidural use in comparison to non-Hispanic Whites [8, 22]. For example, Rust et al. [8] reported lower odds of epidural use among non-Hispanic Blacks when compared to non-Hispanic Whites (OR = 0.49, 95% CI 0.46 - 0.52). It should be noted that study was conducted using Georgia Medicaid recipients rather than all Washington State deliveries.

Several possibilities may explain the associations of epidural use with race and foreign birth. Mistrust of the health care system may result in the decision to forgo epidural analgesia [23]. Provider bias and differences in pain perception have also been presented as possible explanations for these differences [22]. Cultural practices and beliefs may also significantly influence epidural use. In one study, the most common reason provided for declining an epidural was that ‘women should cope with labor pain’, along with family or friends advising against epidural use [24, 25].

Foreign birth, except for women of Asian race, was associated with lower odds of epidural use in our study population. While no prior studies have examined this association directly, a Swedish study found that women born in Asia, Sub-Saharan Africa or Latin America had lower odds of epidural use as compared to Swedish-born women [26]. Though the Swedish study did not stratify by race, that study does indicate a relationship between foreign birth and epidural use.

The lower odds of epidural use among Hispanics, non-Hispanic Blacks, and foreign-born women suggest that additional efforts on the part of health care providers and institutions may be necessary to improve pain management in these populations. Though cultural beliefs, which may contribute to these observed differences, should be respected, it is nevertheless important to ensure equitable access to epidurals and adequate counseling on the risks and benefits of epidural use during labor for all populations.

We speculate that lower odds of epidural use among foreign-born women may also reflect differences in cultural and ancestral backgrounds between foreign-born and US-born individuals. These cultural differences are caused by limited assimilation by foreign-born mothers. Differences in ancestral backgrounds result from the fact that immigrants today, after controlling for race, are from different countries than the ancestors of native-born Washington residents. Furthermore, studies abroad indicate that poor knowledge of epidural analgesia is common [27, 28], and that prior knowledge of epidural analgesia is strongly associated with epidural use [27]. Hence it may be possible that a lack of knowledge about epidurals is partially responsible for lower odds of epidural use for foreign-born populations.

No peer reviewed US studies have investigated the association between hospital perinatal tier and epidural use. However, our finding that perinatal tier is associated with epidural use is consistent with a master’s degree thesis using Washington State birth certificate data from 2003 - 2004 [21]. Our findings could be heavily influenced by the lack of rural hospitals with secondary or tertiary perinatal tiers in our data set. Furthermore, secondary and tertiary perinatal units are designed to handle more complicated births than primary facilities, which themselves are associated with epidural use.

Regarding hospital location, Rust et al. found that women who lived in rural areas of Georgia had lower rates of epidural use (39.2% compared to 62.1% for urban women) [8], which is consistent with our findings. Cultural practices, socioeconomic status, and level of perinatal units present may be causing the observed association.

Understanding the relationship between epidural use and hospital ownership is complicated. For-profit hospitals may be more inclined to support medical interventions, including epidural anesthesia, that would generate revenue. These cost considerations may contribute to increased use of epidurals at these facilities. Additionally, women delivering at for-profit hospitals likely have higher socioeconomic status relative to women at not-for-profit hospitals, which may contribute to the higher odds of epidural use. We report the highest odds of epidural use to be associated with delivering at a military hospital. We speculate that there may be more uniformity in medical practice at military hospitals, which may include the routine utilization of epidurals. Due to the complex nature of this issue, further research is warranted to investigate potential explanations for the observed difference in epidural use among types of hospital ownership. We cannot explain the lower odds of epidural use associated with district hospitals

As epidural use increases, understanding variables that are associated with epidural use is increasingly important. In this study, we have identified several variables associated with epidural use. These associations indicate that disparities in epidural use persist despite its widespread availability, and they underscore the potential clinical importance of epidural use under certain circumstances.
